# TGF-β Physiology as a Novel Therapeutic Target Regarding Autoimmune Thyroid Diseases: Where Do We Stand and What to Expect

**DOI:** 10.3390/medicina57060621

**Published:** 2021-06-14

**Authors:** Efstratios Kardalas, Spyridoula Maraka, Maria Papagianni, George Paltoglou, Charalampos Siristatidis, George Mastorakos

**Affiliations:** 1 Endocrine Unit, Aretaieion Hospital, Medical School, National and Kapodistrian University of Athens, Vasilissis Sofias Str. 76, 11528 Athens, Greece; efkardalas@gmail.com (E.K.); gpaltoglou@med.uoa.gr (G.P.); 2 Division of Endocrinology and Metabolism, Department of Internal Medicine, University of Arkansas for Medical Sciences, 4301 W. Markham St., Little Rock, AR 72501, USA; SMaraka@uams.edu; 3 Unit of Endocrinology, Diabetes and Metabolism, 3rd Department of Pediatrics, Aristotle University School of Health Sciences, Hippokration Hospital of Thessaloniki, Konstantinoupoleos Str. 49, 54642 Thessaloniki, Greece; marpapagianni@hotmail.com; 4 Assisted Reproduction Unit, Second Department of Obstetrics and Gynecology, Aretaieion Hospital, Medical School, National and Kapodistrian University of Athens, Vasilissis Sofias Str. 76, 11528 Athens, Greece; csyristat@med.uoa.gr

**Keywords:** thyroid gland, TGF-β, immune cells, autoimmune thyroid diseases, thyroiditis, pregnancy, therapy

## Abstract

Transforming growth factor beta (TGF-β), as a master regulator of immune response, is deeply implicated in the complex pathophysiology and development of autoimmune thyroid diseases. Based on the close interplay between thyroid autoimmunity and TGF-β, scientific interest was shifted to the understanding of the possible role of this molecule regarding the diagnosis, prognosis, and therapy of these diseases. The main aim of this review is to present research data about possible treatment options based on the role of TGF-β in thyroid autoimmunity. Suggested TGF-β-mediated therapeutic strategies regarding autoimmune thyroid diseases include either the enhancement of its immunosuppressive role or inhibition of its facilitatory role in thyroid autoimmunity. For example, the application of hr-TGF-β can be used to bolster the inhibitory role of TGF-β regarding the development of thyroid diseases, whereas anti-TGF-β antibodies and similar molecules could impede its immune-promoting effects by blocking different levels of TGF-β biosynthesis and activation pathways. In conclusion, TGF-β could evolve to a promising, novel therapeutic tool for thyroid autoimmunity.

## 1. Introduction

The pathophysiology of autoimmune thyroid diseases (Graves’ disease, Hashimoto’s thyroiditis, thyroid-associated orbitopathy, thyroid disease in pregnancy, and post-partum thyroiditis) includes complex immune mechanisms and inflammatory phenomena. Transforming growth factor beta (TGF-β) plays a pivotal role in the proper function of the human immune system and is implicated in the pathophysiological spectrum of thyroid autoimmunity [1,2]. 

Based on the involvement of TGF-β in autoimmune thyroid diseases, it is suggested that novel and reliable prognostic, diagnostic, therapeutic, and follow-up ‘tools’, regarding thyroid autoimmunity, could be developed based on the ‘dual’ immunoregulatory (either suppressive or facilitatory) role of this growth factor. The development of these ‘tools’ entails the fine tuning of the immune effects of TGF-β, in order to avoid adverse effects regarding the ‘smooth’ function of our immune system [3]. Thus, despite increasing scientific knowledge, it remains unclear whether TGF-β could be effectively and safely exploited to change the way patients with autoimmune thyroid diseases are handled.

In this manuscript, the pathways of biosynthesis and activation of TGF-β and its crucial role regarding the immune system and the thyroid physiology are described briefly. Then, the known implications of TGF-β in relation to the diverse pathologic entities of thyroid autoimmunity are presented. Finally, possible diagnostic, prognostic, and therapeutic options based on the involvement of this growth factor in the pathophysiology of thyroid autoimmunity are developed with insistence on treatment options.

## 2. TGF-β Homeostasis and Its Interplay with the Immune System

### 2.1. Physiology of TGF-β Biosynthesis, Activation, and Signaling Pathways

TGF-β is a member of a transforming growth factor superfamily, which includes TGF-βs, bone morphogenetic proteins, activins, and growth redifferentiation factors, among other molecules [4]. ΤGF-β can be found in three isoforms, namely TGF-β1 (the most abundant of the three), ΤGF-β2 and ΤGF-β3. TGF-β is synthesized and secreted by almost all human cells, including all white blood cell lineages. It plays an instrumental regulatory role in the generation, maturation, proliferation, and apoptosis of various cell types; vascular, skeletal, and connective tissue homeostasis; physiological immune response and tolerance; inflammation and fibrosis development; tumor surveillance; and autoimmunity limitation [5,6]. The role of the signaling pathways associated with TGF-β activation and function is fundamental regarding various human cell, tissue, and system properties and homeostasis [7]. 

All TGF-β isoforms are synthesized and produced as biologically inactive molecules, consisting of the pre-TGF-β homodimers [8]. These interact with latency-associated peptides (LAPs), forming a small latent complex (SLC). The latter cannot be released from the cellular environment until it is bound to the latent TGF-β-binding protein (LTBP). The binding of SLC to LTBP induces the ‘construction’ of a much larger complex, which is described as a large latent complex (LLC). During the following step, LLC is secreted into the extracellular matrix (ECM) in an inactive form, which is incapable of binding to TGF-β receptors (TβRs). Only after proteolysis of the LAP from LLC can active TGF-β be released, ready to exert its multifunctional role. The complex multilevel procedure that activates TGF-β is mediated by a variety of diverse factors, such as metalloproteases, integrins, thrombospondin-1, pH, and reactive oxygen species including hydroxyl radicals’ reactive oxygen species [9]. 

The majority of human cells express three types of TβRs [10]. TβRI and TβRII are transmembrane serine/threonine kinase receptors, while TβRIII is mainly a type I integral membrane protein co-receptor. Initially, TGF-β binds to TβRII (under the mediation of TβRIII) resulting in the recruitment of TβRI. As a result, heteromeric activation complexes are formed on cell surface, comprising two pairs (dimerization) of TβRI and TβRII, respectively, with each pair binding one of the two chains of active TGF-β. Within this activation complex, TβRII phosphorylates/activates TβRI, thus initiating the cascade associated with TGF-β intracellular signaling [11]. Until now, only some parts of the TGF-β intracellular signaling pathways are decoded, while the full mechanisms remain unclear. A number of these TGF-β activating pathways are unique for distinct cellular/tissue types, while others can be traced to a broad range of different cell/tissue categories [12]. The intracellular signaling pathways of TβRs are mediated by eight structurally resembling proteins, the so-called Smads (homologies to the Caenorhabditis elegans SMA (“small” worm phenotype) and MAD family (“Mothers against Decapentaplegic”) of genes in Drosophila). Hence, the Smad-dependent (or canonical) pathway is the primary signaling pathway for TGF-β [13]. In particular, after TβRI is phosphorylated, it can induce downstream Smad 2 and 3 (R-Smads) phosphorylation. Directly after, the binding of phosphorylated R-Smads to the common-partner Smad4 (Co-Smad) takes place, leading to the synthesis of the R-Smads/Co-Smad complex. Following its nuclear translocation, this complex modulates the transcriptional process of TGF-β target genes, which, regarding T-cells, are still not fully specified. Moreover, transcriptional intermediary factor 1γ (TIF1γ) has the ability to bind to the phosphorylated Smad2/3 complex (Smad binding complex or SBE), thus presenting with an antagonizing action to that of Smad4 [14]. Furthermore, inhibitory Smad6 and Smad7 (I-Smads) can also exert a regulatory role regarding TGF-β signaling via negative feedback. The amounts of I-Smads are TβR-dependent and increase in parallel with the intensification of the TGF-β signaling cascade activity [15]. Moreover, it has been shown that a clear association between the TGF-β-mediated suppression of cell proliferation and the Smad-dependent (or canonical) pathway exists [16].

To the contrary, the non-Smad-dependent (or non-canonical) signaling pathway is mediated by the recruitment of Smad7 to the complex comprising either phosphorylated Smad2/3C or activated ΤβRs [17]. Regarding the Smad-independent signaling pathway, accumulating research data have established the significance of various contributing factors for the uninterrupted and effective signal transmission of the TβR activation complex [18]. More precisely, factors such as TGF-β-activated kinase 1 (TAK1), p38 mitogen-activated protein kinase (p38 MAPK), extracellular signal regulated kinase (ERK), tumor necrosis factor (TNF), or nuclear factor kappa B (NF-κB) enhance or inhibit the cellular response to non-canonical TGF-β signaling pathways [19]. The dysregulation of both the canonical and non-canonical TGF-β signaling pathways is directly or indirectly implicated in several pathological processes, including autoimmune and inflammatory disorders.

### 2.2. The Physiological Role of TGF-β Regarding the Immune System

Innate (or natural) and adaptive (or active) immunity are the two main ‘arms’ of the human immune system [20]. Innate immunity is a born immune antigen non-specific defense mechanism, which is mediated by a variety of immune cells, including macrophages, dendritic cells, neutrophils, monocytes, and mast cells. Interestingly, cell types involved in the innate immune system catalyze the development of adaptive (or acquired) immunity, which is antigen-specific and evolves throughout the human life. The adaptive immune response can be further subdivided in humoral and cell-mediated, which is defined by the involvement of B- and T-cells, respectively [21]. The interplay of TGF-β with both components of the human immune defense mechanisms is complex. Regarding innate immunity, this growth factor modulates the homeostasis and exerts both promoting and suppressing effects on cellular life and function [22]. 

Equivalently, TGF-β is implicated in the adaptive T-cell immunity either in an immune-enhancing or immune-suppressive manner [23]. Concretely, T-cell physiology and activity is substantially modulated by TGF-β. The role of this molecule is vital regarding every aspect of the synthesis, differentiation, maturation, activation, survival, and destruction of T-cells. Furthermore, TGF-β regulates the immune effects and the regulatory mechanisms, which are catalyzed by T-cells (Table 1). The effects of TGF-β on T-cell homeostasis are regulated by factors associated with and regulated by the extra- and intracellular microenvironment [23,24].

In addition to T-cells, B-cells comprise the other important cell population involved in the adaptive immune mechanism [21]. After their development in hematopoietic stem cells, B-cells migrate to the secondary lymphoid organs, where they can be activated, either independently or following interaction with T-cells [25,26]. Antibody secretion is the vital function of B-cells regarding the adaptive humoral immunity. Therefore, it is clear that the role of TGF-β in B-cell physiology is equally important to that in T-cell physiology (Table 2). 

Conclusively, almost the whole spectrum of immune system functions is affected by TGF-β activity. Deeper understanding of the specific factors that stimulate TGF-β secretion and regulate its function is needed to decode the ‘hidden’ immune pathways and ‘neutralize’ or even reverse the destructive consequences of autoimmune disorders.

## 3. Thyroid Gland Homeostasis and TGF-β

### 3.1. Interplay between Normal Thyroid Physiology/Function and TGF-β

A delicate modulation of the growth and proliferation of thyroid follicular cells coordinates and controls the thyroid gland homeostasis and function. The former depends, among others, on various growth factors, including those comprising the superfamily of transforming growth factors and especially TGF-β [27]. Thyroid follicular cells proliferate and differentiate tightly controlled by (a) TSH (thyroid stimulating hormone), which plays a systemically stimulating role and (b) locally produced TGF-β, which exerts an inhibitory paracrine function. More specifically, TGF-β has a major regulatory role in thyroid physiology. More specifically, it inhibits mainly the proliferation and limits the function of thyroid follicular cells. Interestingly, sex steroids modulate intrathyroidal TGF-β synthesis and function [23]. Estrogens activate TGF-β signaling via Smad 2 phosphorylation, while estrogenic effects on thyroid follicular cells are mediated by intrathyroidal produced and secreted TGF-β [28]. It is also known that thyroid follicular cells express both types of estrogen receptors (ΕRs; ER-α and ER-β) [29]. Interestingly, TGF-β inhibits ERs expression via Smad4. In addition, the activation of ER-β triggers a sequence of TGF-β-regulated actions, resulting in Th17 type responses, while the activation of ER-α prompts Smad2/3 proteins deficiency or depletion, thereby impeding TGF-β signal transduction [30,31]. On the other hand, TGF-β transcriptional responses can be minimized or inhibited via androgen-regulated obstruction of the attachment of Smad3 to the Smad binding complex (or SBE) [32]. Likewise, TGF-β can be impeded by progesterone. This hormone is an antagonist of TGF-β regarding its function on Smad activation and inhibits TGF-β/Smad-induced signaling and transcription pathways [33]. 

### 3.2. Association of TGF-β with the Pathophysiology of the Autoimmune Thyroid Diseases

Thyroid autoimmunity development is a result of combined genetic predisposition and immune mechanisms dysregulation. The vital role of TGF-β regarding the pathophysiology of autoimmune thyroid diseases is supported by scientific data, which indicate that this growth factor affects in a disease type-specific manner the intractability, severity, gravity, and/or course of each one of the autoimmune thyroid diseases [1,2]. The exact role of TGF-β (either inhibitory or facilitatory) regarding thyroid autoimmunity depends largely on the synthesis rate of this growth factor, the unique genotypic and phenotypic characteristics of the specific autoimmune thyroid disease, and the activation of distinct signaling pathways. In the following paragraphs, the role of TGF-β in different autoimmune thyroid diseases is briefly discussed. More specifically:

#### 3.2.1. In Graves’ Disease

**Figure 1:**
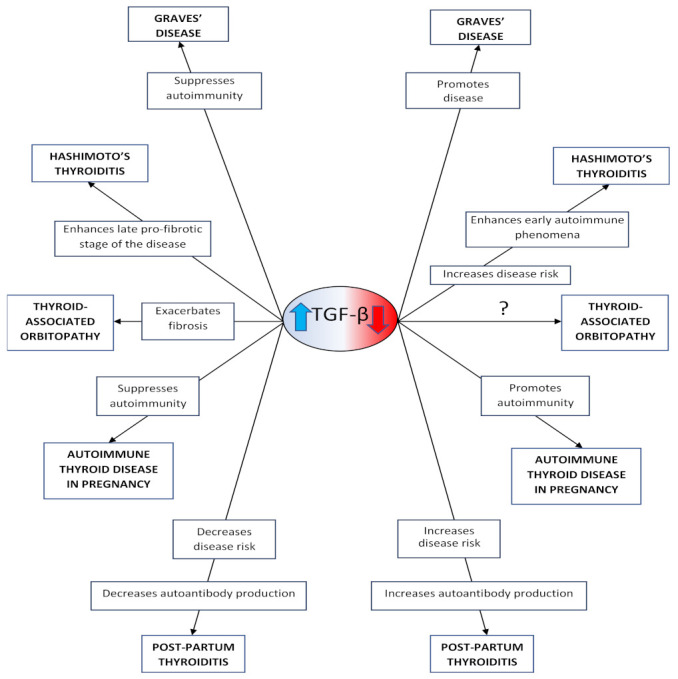
Implication of TGF-β in the development of the autoimmune thyroid diseases.

TGF-β deficiency contributes to its pathogenesis via increase of the IFN (interferon) γ-induced expression of MHC (Major Histocompatibility Complex) class II antigens; increased T-cell generation, maturation, and differentiation; alteration of Th1 cell and Th2 cell-regulated responses; enhanced B-cell production and antibodies synthesis; inhibition of the suppressive activity of Tregs (T regulatory cells) [34,35] (Figure 1). Interestingly, the depletion of T-regulatory cells in rodents renders the latter increasingly susceptible to experimental Graves’ disease [36]. Experimental administration of human recombinant TGF-β (hrTGF-β) to patients with Graves’ disease or to thyroid follicular cell/lymphocyte co-cultures constrains the proliferation of lympho- and monocytes in the periphery as well as of T-cell clones either in the periphery or in the thyroid. Furthermore, the above-mentioned hrTGF-β administration suppresses the T-cell clones-specific mechanisms, which allow them to recognize thyroid follicular cells and limit the expression of TPO and MHC class II autoantigens [37]. Moreover, the genetic polymorphisms in the TGF-β gene (at codons 10 and 25), which are associated with reduced serum TGF-β concentrations, increase substantially the risk for Graves’ disease development [38]. 

#### 3.2.2. In Hashimoto’s Thyroiditis

The interplay between Th cells and thyroid-specific autoantibodies (ATA) is regulated by TGF-β, which exerts a flexible role in the pathophysiology of Hashimoto’s thyroiditis. This role depends exclusively on the pathophysiologic stage of the disease [39]. Hashimoto’s thyroiditis is associated at its initial pathophysiological stage with diminished serum TGF-β concentrations, which generate a significant modification of Th1 and Th2 cell-regulated immune functions and inhibition of the physiologic suppressive Tregs activity [40] (Figure 1). Moreover, the activation of TGF-β-producing iTregs has been shown to avert the development of experimental autoimmune thyroiditis (EAT) [41]. In experimental rodent models, specific, transgenic enhancement of a selective cytotoxic T-lymphocyte antigen 4 (CTLA-4) led to expanded i (induced) Tregs synthesis, which is accompanied by increased TGF-β production and, subsequently, the limitation of Hashimoto’s thyroiditis evolution. At the final pathophysiologic stage, increased serum TGF-β concentrations stimulate autoreactive T-cells synergistically with B-cells and antibodies to induce an extensive depletion and apoptosis of thyroid follicular cells, leading to the development of hypothyroidism [40,42]. Thus, TGF-β exerts a ‘dual’ role regarding Hashimoto’s thyroiditis, depending on the pathophysiologic stage of the disease (initially suppressing autoimmunity and at the end probably stimulating fibrosis) [43]. Genetic polymorphisms of the TGF-β gene (at codon 10) are associated with decreased serum TGF-β concentrations as well as a significantly increased risk for and enhanced susceptibility to Hashimoto’s thyroiditis [44]. 

Abnormal TGF-β serum concentrations play a vital role in the pathophysiology and development of autoimmune thyroid diseases. Increased serum TGF-β concentrations have many consequences: suppression of the development of Graves’ disease; enhancement of the final pro-fibrotic stage of Hashimoto’s thyroiditis; exacerbation of fibrosis in thyroid-associated orbitopathy via enhanced maturation and differentiation of myofibroblasts, hyaluronan acid synthase activity, and cytokine synthesis; and suppression of autoimmune thyroid disease in pregnancy. Furthermore, they are also associated with decreased risk for post-partum thyroiditis and decreased autoantibody production. Meanwhile, decreased serum TGF-β concentrations promote the expression of Graves’ disease; increase susceptibility to Hashimoto’s thyroiditis and trigger the autoimmune phenomena during its initial stage; promote autoimmunity in autoimmune thyroid disease in pregnancy; and are associated with increased risk for post-partum thyroiditis and increased autoantibody production.

#### 3.2.3. In Thyroid-Associated Orbitopathy

Thyroid-associated orbitopathy develops via complex inflammatory procedures and dysregulated immune mechanisms, which are tightly controlled by TGF-β. This molecule is involved in the development of immune tolerance against self-antigens but can also promote the maturation and differentiation of orbit-localized myofibroblasts via increased production of hyaluronic acid (HA) 1 and 2 [45]. Following TGF-β stimulation, orbital fibroblasts that express CD90 differentiate into myofibroblasts [46]. In patients with thyroid-associated orbitopathy, TGF-β stimulates myofibroblast proliferation and the synthesis of cytokines and hyaluronic acid synthase (HAS), while it contributes to TSHr (TSH receptor) degradation and to the deceleration of adipogenesis [47] (Figure 1). In addition, TGF-β enhances sphingosine-1-phosphate (S1P) expression, which is a significant positive regulator of fibrosis in case of thyroid-associated orbitopathy, thus promoting ocular fibrosis [48]. Moreover, serum TGF-β concentrations regulate HAS production from orbital fibroblasts, while ECM expansion is stimulated via TGF-β [49]. It is interesting that experimental treatment of ocular fibroblasts and orbital tissue (extracted from patients with thyroid-associated orbitopathy) induced an increased expression of plasminogen activator inhibitor 1 (PAI-1), which is a serine protease inhibitor functioning as a downstream effector of the fibrotic response [50]. To be precise, PAI-1 limits ECM degradation and bolsters tissue fibrosis as well as the endurance and gravity of the inflammatory response. The exogenous delivery of PAI-1 increases serum TGF-β concentrations in several cell types, indicating the presence of a PAI-1/TGF-β-positive feedback mechanism and a latent, not fully understood pro-fibrotic interplay. Additionally, unique polymorphisms of the TGF-β genetic pool (especially at codons 10 and 25) significantly multiply the possibility for thyroid-associated orbitopathy development [51]. 

#### 3.2.4. In Autoimmune Thyroid Diseases during Pregnancy

TGF-β physiologically exerts an important autocrine, paracrine, and endocrine role during pregnancy [52]. Maternal serum TGF-β concentrations increase significantly during the gestational period in comparison to the non-gestational period as established in experimental rodent models. Moreover, maternal serum TGF-β concentrations are lower in the third than in the first and second trimester of pregnancy (Figure 1). Indeed, our recent research showed that serum TGF-β concentrations are higher during the 24th gestational week and the 1st post-partum week compared to those in the 36th gestational week [53]. In that study, mean serum TGF-β concentrations at the observation time-points evolved in a “mirror” image fashion in regard to the correlative mean serum cortisol concentrations, strongly indicating the existence of an important, tight physiologic interplay between TGF-β and glucocorticoids during gestation. It is noteworthy that the addition of TGF-β1 to human adrenocortical tumor cell cultures has resulted in a significant inhibition of adrenocortical cell steroidogenesis, leading to the hypothesis that TGF-β1 can suppress cortisol production via the decrease of both the cytochrome P450 family 11 subfamily B member 1 (CYP11B1) mRNA levels and steroid 11β-hydroxylase activity [54]. Furthermore, regarding autoimmune thyroid diseases during pregnancy, the rise of serum TGF-β concentrations observed during their toxic phase indicates that the interplay of immune mechanisms/inflammation and pregnancy could stimulate and accelerate the activation of this important growth factor [55]. 

##### 3.2.5. Ιn Post-Partum Thyroiditis

TGF-β can ‘normally’ avert the development of post-partum thyroiditis. More specifically, this molecule suppresses the development of thyroid autoimmunity in the post-partum period by facilitating the transformation of naïve Tregs to iTregs, thus promoting their immunosuppressive role [55] (Figure 1). Based on our recent study, we reported that the difference between the serum TGF-β concentrations at the 36th gestational week and the ones at the 1st post-partum week (decreased and increased respectively) has an important negative predictive value regarding post-partum anti-TPO antibodies concentration [53]. Thus, this observation implies a significant, direct, and/or indirect inhibitory immune effect of TGF-β regarding the rise of anti-TPO antibodies concentrations associated with the post-partum period. Moreover, serum TGF-β concentrations are reduced between the first transitory hyperthyroid phase (during which this growth factor is intrathyroidal produced and subsequently activated, stimulating thus thyroid follicular cell destruction, and increasing in an autocrine manner its own synthesis) and the second (associated with hypothyroidism) phase of post-partum thyroiditis [55]. The evolution of the latter and serum TGF-β concentrations seem to interrelate closely. Thus, this growth factor could potentially be used as a novel, reliable predictive marker of the progression toward the confinement and cure of post-partum thyroiditis [56]. Increased serum TGF-β concentrations may catalyze an elaborate intrinsic, but only partially understood, mechanism, which suppresses inflammatory phenomena and could ultimately prevent permanent immunological damage of the thyroid gland during the post-partum year. 

In conclusion, although the importance of TGF-β regarding thyroid autoimmunity is undisputed, the role of this growth factor in the development of the autoimmune thyroid diseases needs further research. On one hand, decreased serum TGF-β concentrations have the following effects (Figure 1):

- Contribute substantially to the pathophysiology of Graves’ disease [2], while they correlate negatively with the difficulty of treating this condition [38].
- Increase the susceptibility to Hashimoto’s thyroiditis [41] and trigger the immune phenomena defining the initial pathophysiologic stage of this disease [42].
- Are associated with increased risk for post-partum thyroiditis [53], prolonged hyperthyroid phase, and increased autoantibody production in this disease [55].

On the other hand, increased serum TGF-β concentrations have the following effects (Figure 1):

- Enhance the final pro-fibrotic stage of Hashimoto’s thyroiditis [43]
- Exacerbate fibrosis in thyroid-associated orbitopathy via enhanced myofibroblast maturation and differentiation and expanded HAS and cytokine synthesis [47,48,49].

## 4. TGF-β-Based Therapeutic Interventions in Non-Thyroid Autoimmune, Inflammatory, and Fibrotic Diseases

The therapeutic approaches implicating TGF-β and its complex physiology can be differentiated in those exploiting the immune-suppressive and those based on the immune-promoting properties of this molecule regarding the generation of certain conditions.

The immune-suppressive role of TGF-β is exploited in the following conditions:

- Experimental addition of iTregs (generated in healthy subjects) to patients with active multiple sclerosis effectively inhibited the autoimmune phenomena associated with the latter via enhanced TGF-β synthesis [57].
- In arteriosclerotic conditions (especially in atherosclerosis), TGF-β has a highly beneficial and protective role, as it inhibits the migration of macrophages and suppresses their transformation to smooth muscle cells; sustains and maintains the normal function of the endothelium; minimizes the adhesiveness of the latter regarding pro-inflammatory cell types; and protects vascular integrity [58]. Thus, decreased serum TGF-β concentrations correlate with greater cardiovascular death risk. As a result, therapeutic strategies involving TGF-β could be extremely useful toward ischemic heart disease prophylaxis and limitation of cardiovascular mortality.

To the contrary, suppression of the immune-facilitating role of TGF-β is exploited in specific conditions. The development of pioneering treatments is in progress, targeting the inhibition of TGF-β endogenous production, activation, and signaling pathways, and counterbalancing adequately the ensuing immune functions of this molecule:

- Regarding fibrosis, gene transfer of Smad7 (which exerts a suppressive role in TGF-β signaling pathways) has been therapeutically applicated to liver and colon fibrosis, diabetic nephropathy, and vascular smooth cell remodeling [59].
- PPARγ (Peroxisome proliferator-activated receptors-γ) agonists limit fibrosis in liver via mechanisms unknown yet. Eventually, PPARγ agonists inhibit TGF-β-mediated activation of TβRI signaling in hepatic stellate cells, thereby effectively inhibiting Smad3-dependent initiation of ECM gene expression, which leads to substantially decreased PAI-1 and collagen-1α synthesis [60]. The experimental treatment of these cells with PPARγ agonists results in a dose-dependent suppression of TGF-β-mediated functions, preserving at the same time the physiological cell development or survival.
- In many diseases such as alcoholic steatohepatitis, sarcoidosis, post-radiation fibrosis, and various types of neuropathy, the suppressed expression of TGF-β restores the disturbed Th1/Th2 equilibrium and limits Th1-regulated immune responses. Pentoxifylline (PTX) has been therapeutically used to effectively suppress TGF-β activation and prevent the detrimental autoimmune responses associated with this growth factor [61].
- Treatment strategies based on progesterone had been effective in treating preterm infants suffering from bronchopulmonary dysplasia via dose-dependent suppression of TGF-β-mediated activation and ensuing transcription of the Smad2/3 complex [33].
- Suppression of TGF-β activation and/or signaling pathways via small peptides with binding affinity to the LAP region of the TGF-β latent complex can adequately block TGF-β induced fibrosis and thus promote liver regeneration in experimentally hepatectomized rodents [62].

It is conceivable that these or similar therapeutic regimens could be tested in treating thyroid autoimmune conditions.

## 5. Suggested TGF-β-Based Diagnostic, Prognostic, and Therapeutic Interventions in Thyroid Autoimmune Diseases

Serum TGF-β concentrations either alone or together with other markers, such as ATA (antithyroid antibodies), could be used for diagnostic, prognostic, and therapeutic follow-up purposes regarding thyroid autoimmunity. Particularly, serum TGF-β concentrations are proposed to be useful and reliable markers for punctual diagnosis and adequate therapy initiation in case of patients who present with non-typical symptoms of hyperthyroidism and are susceptible to Graves’ disease [63]. Furthermore, the above-mentioned markers could be used for the prognosis of fibrosis in thyroid-associated orbitopathy and the effective follow-up of the therapeutical approaches of this disease [64]. Finally, increased third gestational trimester serum TGF-β concentrations might correlate to greater hazard for the development of autoimmune thyroid diseases during the post-partum period. [53]. 

Direct and/or indirect implication of TGF-β in the complex pathophysiological mechanisms of thyroid autoimmunity has led to extended scientific research targeting the homeostasis of this growth factor and aims at the development of novel TGF-β-mediated therapeutic interventions. Treatment strategies regarding this growth factor could be applied in various intra- and extracellular levels of TGF-β biosynthesis, activation, activity, and transcription. Nonetheless, so far, no TGF-β-oriented treatments (regarding thyroid autoimmunity) have been granted official approval by the European Medicines Agency or the U.S. Food and Drugs Administration. Research efforts intending to develop such treatments should take notice of the complexity/duality of the TGF-β role regarding the autoimmune thyroid diseases (immune-inhibitory or -facilitating) 

The following apply in relation to the inhibitory role of TGF-β in immune responses:

- Treatment of human cultures of thyroid follicular cells/lymphocytes (from Graves’ disease patients) with hrTGF-β limits autoimmune phenomena in the thyroid gland via substantially diminished target T-cells antigenicity and decreased expression of TPO and MHC class II autoantigens [37].
- Treatment with low-level laser therapy (LLLT) exerts a suppressive role regarding the evolution of autoimmune diseases via the regeneration of various tissues. It has been shown that in case of Hashimoto’s thyroiditis, treatment of patients with LLLT leads to a significant stimulation of TGF-β gene expression [65]. Thus, this therapeutic strategy promotes normal thyroid function, which is ‘mirrored’ in the mean daily levothyroxine dose-reduction. As a result, therapeutically applicated LLLT to Hashimoto’s thyroiditis could be an effective tool of autoimmunity restraint during the initial pathophysiologic stages of this disease via enhanced TGF-β biosynthesis and subsequent cytokine gene expression.

The following apply in regard to the immune-facilitating properties of TGF-β:

- The limitation of the Smad-regulated TGF-β gene transcription can substantially suppress thyroid gland fibrosis, which is observed in animal models of experimentally induced autoimmune thyroiditis [66]. This limitation can be accomplished following the administration of triiodothyronine (T3), which binds to its thyroid receptors complex and represses specific TGF-β activated gene promoters.
- The fibrotic phenomena observed in the final pathophysiologic stage of Hashimoto’s thyroiditis can be limited in animal models of this disease via monoclonal anti-TGF-β antibodies administration, which neutralizes the excess quantities of extracellular TGF-β [67].
- The application of 17-estradiol (E2) to thyroid follicular cells (rodent models) triggers ER-β-mediated development of EAT [28]. To the contrary, treatment with an E2 antagonist, such as coumestrol, limits ATA production and suppresses TGF-β-induced Th17-type response (which promotes “cell-type” autoimmune response) [29]. It should be noted that ER-α activation can counterbalance the outcomes of TGF-β function in a unique E2-dependent manner [30,31].
- In Hashimoto’s thyroiditis, TGF-β-mediated inflammatory phenomena initiate enhanced cyclooxygenase (COX-2) expression [68]. Thus, suppression of the latter via Celecoxib (an inhibitor of COX-2) might be an effective treatment strategy for the limitations of the autoimmune process in Hashimoto’s thyroiditis.
- The addition of Celecoxib to human myoblasts (cultured from extraocular muscles of patients with thyroid-associated orbitopathy) impedes TGF-β-regulated HA production and disrupts the TGF-β-mediated maturation and differentiation of ocular muscle fibroblasts [68].
- The addition of PPARγ agonists (such as pioglitazone) to above-mentioned human myoblasts can restrain inflammatory phenomena and suppress adipogenesis in orbital fibroblasts [68]. Moreover, PPARγ agonists can inhibit the facilitatory role of TNF regarding TGF-β-biosynthesis via canonical and/or non-canonical TGF-β-related signaling pathways. Consequently, the catalyzing effects of TGF-β regarding (a) myofibroblast formation from fibroblasts and (b) HAS, HA, and TGF-β production in retro-orbital fibroblasts are adequately limited in an indirect, paracrine manner.

Thus, it has been proven that in thyroid-associated orbitopathy, these two pharmaceutical agents (COX2 inhibitors and PPARγ agonists) can substantially limit TGF-β-mediated chemokine and cytokine synthesis via disruption of TGF-β activity and signaling.

- Furthermore, aryl hydrocarbon (AH) can suppress the profibrotic activity of TGF-β in thyroid-associated orbitopathy [69]. Thus, AH agonists or AH receptor ligands inhibit TGF-β mediated myofibroblast formation in patients with thyroid-associated orbitopathy via the blockade/attenuation of the profibrotic Wnt signaling.
- In addition, the stimulatory effect of PAI-1 regarding TGF-β production in patients with thyroid-associated orbitopathy can be reversed via synthetic or natural small molecules, which effectively suppress the function/activity of PAI-1 [70]. The therapeutic application of these molecules regarding PAI-1 has already been initiated with success in fibrotic, malignant, and thromboembolic conditions.

In conclusion, despite the fact TGF-β-oriented treatments regarding thyroid autoimmunity are still at an experimental level, targeting the dual immune role of TGF-β (inhibitory or enhancing) could lead to the development of innovative therapies for autoimmune thyroid diseases (Table 3).

## 6. Conclusions

TGF-β plays a vital role regarding the integrity of the immune system and the physiology of the thyroid gland. Furthermore, this molecule is deeply implicated in the generation and development of thyroid autoimmunity. Extensive research data illustrate a potent role of TGF-β regarding the diagnosis and prognosis of thyroid autoimmunity. Moreover, TGF-β could be used toward the development of novel treatment modalities for autoimmune thyroid diseases. Even though these modalities are still at an experimental level, targeting the dual immune role of TGF-β (inhibitory or enhancing) could lead to the development of innovative therapies for autoimmune thyroid diseases that are conditioned on the subtype and the distinct pathophysiology of the latter. Such treatment modalities could restrain inflammatory phenomena; inhibit thyroid follicular cells depletion; limit substantially the thyroid gland infiltration with leucocytes; initiate intensified regenerative mechanisms of dysfunctional thyroid follicular cells; and suppress intrathyroidal and intraorbital fibrotic processes, which are all phenomena associated with the pathophysiology of autoimmune thyroid diseases. The success of treatment strategies involving TGF-β in case of other autoimmune pathologic conditions supports the theory that the development of similar therapies could have beneficial effects on autoimmune thyroid diseases. Of course, the idea of novel, TGF-β oriented therapies is fascinating but is not free of substantial dilemmas and hazards, as a longstanding suppression or enhancement of TGF-β activation and signaling pathways can disrupt multiple cellular functions and physiological tissue mechanisms. 

In conclusion, TGF-β is a key transforming growth factor, which has an exceptional, unparalleled, ‘dual’ immunoregulatory effect (either inhibitory or facilitating) regarding thyroid autoimmunity. A deep and thorough interpretation of the complex involvement of TGF-β in autoimmune thyroid diseases pathophysiology could trigger more focused research for the development of diagnostic and prognostic tools. Avant-garde techniques could modify TGF-β synthesis and activity, resulting in novel treatment options for autoimmune thyroid diseases. 

## Figures and Tables

**Table 1:** Effects of TGF-β in Τ-cell physiology and development.

TGF-β
Stimulates naive CD4+ T-cells transformation to effector T-cells Suppresses the proliferation and differentiation of effector T-cells via inhibition of Th2-produced IL-2 Alters the type of produced cytokines and mediates phenotypic metamorphosis among effector T-cells Enhances TNF production by both CD4+ and CD8+ T-cells Enhances the proliferation of CD8+ cells (in experimental mouse models) Stimulates transformation of nTregs to iTregs via increased Foxp3 expression Promotes Treg-induced inhibition of the exocytosis of granules Inhibits the generation and activation of CTLs Suppresses the cytotoxicity of the CTLs via the transcriptional regression of genes encoding proteins, which are vital for CTLs function

**Table 2:** Effects of TGF-β in B-cell physiology and development.

TGF-β
Is secreted by B-cells (which express its receptors) Inhibits B-cell activation and antibodies production Promotes class switching of IgA in both human and mouse B-cells Inhibits immunoglobulin synthesis and class switching to the majority of IgG isotypes Induces apoptosis of immature or resting B-cells by an unknown yet mechanism, which may overlap with its anti-proliferation pathway.

**Table 3:** Experimental therapeutic applications that target TGF-β physiology (synthesis, activation, action) in autoimmune thyroid diseases.

	Effect on Synthesis	Effect on Activation	Effect on Action	Experimental Therapeutic Application
Exogenously administrated hr-TGF-β	None	None	None	Cultures of follicular thyroid/lymphocyte cells from Graves’ disease in humans
Low-level laser therapy	Increase	None	None	Hashimoto’s thyroiditis in humans
Small peptides	None	Inhibit TGF-β disengagement from LAP	None	Cancer animal models
Monoclonal anti-TGF-β	None	None	Neutralize excess extracellular TGF-β	Hashimoto’s thyroiditis in animal models [61]
Triiodothyronine nuclear receptor ligands	None	None	Limit Smad phosphorylation	Thyroid fibrosis in animal models
Estrogen receptor β antagonists	None	None	Inhibit TGF-β-mediated Th17-type response	Experimental autoimmune thyroiditis in animal models
Estrogen receptor α agonists	None	None	Suppress TGF-β activity	Experimental autoimmune thyroiditis in animal models
COX-2 inhibitors	None	None	Block TGF-β-induced HA synthesis. Decrease TGF-β-induced ocular muscle fibroblasts proliferation	Cultures of extraocular muscle fibroblasts from *TAO* in humans
PPAR-γ agonists	Inhibit TNF- mediated TGF-β synthesis	None	Inhibit TGF-β-induced fibroblast differentiation to myofibroblasts.Decrease HAS and HA synthesis	Cultures of extraocular muscle fibroblasts from *TAO* in humans
